# *Rhodnius prolixus* Colonization and *Trypanosoma cruzi* Transmission in Oil Palm (*Elaeis guineensis*) Plantations in the Orinoco Basin, Colombia

**DOI:** 10.4269/ajtmh.19-0331

**Published:** 2020-05-26

**Authors:** Diana Erazo, Camila González, Felipe Guhl, Juan Daniel Umaña, Juan Alejandro Morales-Betancourt, Juan Cordovez

**Affiliations:** 1 Grupo de Investigación en Biología Matemática y Computacional (BIOMAC), Universidad de los Andes, Bogotá, Colombia;; 2 Centro de Investigaciones en Microbiología y Parasitología Tropical (CIMPAT), Facultad de Ciencias, Universidad de los Andes, Bogotá, Colombia

## Abstract

*Trypanosoma cruzi* is the etiological agent of Chagas disease that infects more than seven million people in Latin America. The parasite is transmitted by triatomine insects, of which some species are often associated with palms. The establishment of oil palm plantations (*Elaeis guineensis*) in the Orinoco region (Colombia) has been rapidly growing, possibly constituting a new environment for the establishment and increase in triatomine populations. In this study, the potential of *Rhodnius prolixus* to colonize *E. guineensis* plantations and maintain *T. cruzi* transmission was assessed. Fieldwork was conducted in two areas located in the department of Casanare for sampling *E. guineensis* and *Attalea butyracea* palms, sampling for triatomines to determine their abundance and prevalence of *T. cruzi* infection. To assess *T. cruzi* transmission potential in the area, sylvatic and domestic mammals were sampled. Results showed that palm infestation with triatomines was higher in *A. butyracea* than in *E. guineensis* palms and *T. cruzi* infection in triatomines varied between habitats for one study area, but was constant in the other site. *Trypanosoma cruzi*–infected mammals in the *E. guineensis* plantations were mainly generalist rodents, suggesting that these mammals could have an important role in *T. cruzi* transmission in plantations. In conclusion, *E. guineensis* plantations in the Orinoco region are suitable habitats for *R. prolixus* and *T. cruzi* transmission.

## INTRODUCTION

In 2012, the WHO included Chagas disease in the 2020 goals program for controlling the burden of morbidity of neglected tropical diseases (NTDs).^1^ Chagas disease is an NTD in Latin America with a burden of 10,000 deaths per year.^2^ The disease is caused by the parasite *Trypanosoma cruzi* and transmitted to humans and other mammalian hosts by several insect species of the Triatominae subfamily. In Colombia, recent estimates suggest that 1% of the human population is infected and 15% is at risk.^3^ Without treatment, Chagas disease can cause serious heart complications leading to death.

*Rhodnius prolixus* is the main vector species in Colombia.^4^ The natural habitat of *Rhodnius* species are large-crown palms, particularly *Attalea butyracea*, which are ubiquitous in the department of Casanare, Orinoco region.^5,6^ *Rhodnius. prolixus* has also been collected in human dwellings, but colonization remains a matter of debate in Colombia and Venezuela because of reinfestation by sylvatic populations.^6,7^ Because *R. prolixus* is capable of invading oil palms (*Elaeis guineensis*),^8^ the establishment of these plantations in the region imposes a risk and a possible new transmission scenario.

Oil palm plantations (*E. guineensis* Jacq: Arecaceae), originally from west and southwest Africa, were established in Colombia during the 1960s.^9^ Currently, *E. guineensis* is a commonly used crop for biodiesel production in the world, and Colombia is the main producer in Latin America and the fourth in the world. National policy actively promotes expansion of such crop production for increased contribution to the future of the biodiesel market.^10^ In the Orinoco region, the annual area devoted to the plantation increases at a rate of 7,396.3 ha/year,^11^ being one of the most noticeable land use alterations and replacing to a great extent the natural savanna.^11^

Land use change has been suggested as major driver of the emergence of infectious diseases.^12^ In particular, *E. guineensis* plantation establishment has the potential to significantly impact transmission of vector-borne diseases.^11,13,14^ For instance, *E. guineensis* development in Malaysia has been linked to scrub typhus because plantations promote colonization of typhus vector mites and rodent hosts.^13^ In Papua New Guinea, *E. guineensis* plantations have been associated with dengue cases, as the plantations provide breeding sites for mosquitoes during the rainy season.^14^

*Elaeis guineensis* palms have large crowns suitable for triatomine colonization, similar to *A. butyracea*, and the plantations could harbor mammal species, supporting *T. cruzi* transmission. Nonetheless, *E. guineensis* plantation establishment represents the introduction of a new environment in the region, composed by a single plant species. By contrast, *A. butyracea* palms are typically found in gallery forests that comprise several tree species. *Elaeis guineensis* plantations could be increasing the risk of Chagas disease transmission in Colombia; however, the long-term impact of these plantations on Chagas disease infection risk in humans has not been fully evaluated.^11^ The main objective of this study was to assess the potential of *R. prolixus* populations to colonize *E. guineensis* plantations and maintain *T. cruzi* transmission in two study sites in the department of Casanare, Colombia. In addition, sylvatic and domestic mammals were tested for *T. cruzi* infection in these two localities. Finally, results were compared between *E. guineensis* and *A. butyracea* habitats.

## MATERIALS AND METHODS

### Ethics statement.

Sampling procedures were performed with Universidad de Los Andes collection approval (Resolution 1177 October 9, 2014—IDB 0359) from the National Authority of Environmental Licenses and the ethical review board of the Universidad de los Andes (CICUAL–C.FUA 14-026).

### Study sites.

**Figure 1.**
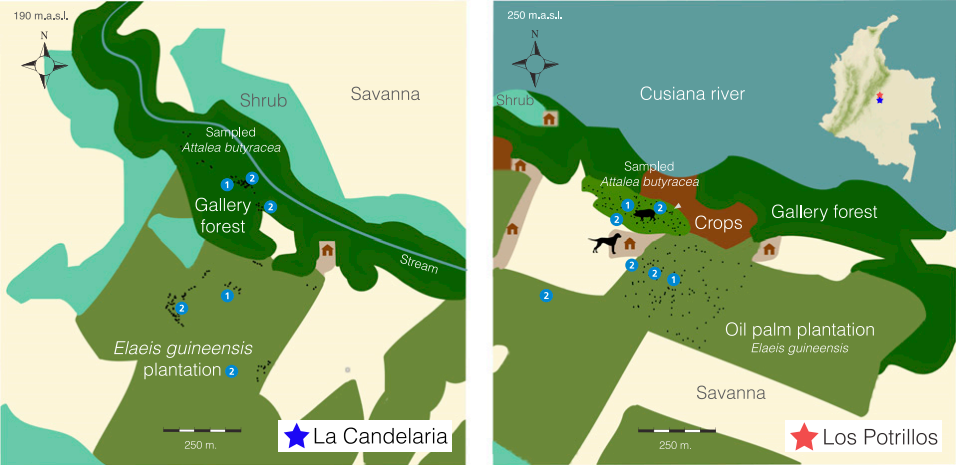
Study sites—La Candelaria and Los Potrillos (LP). Study sites are located in the municipality of Tauramena, Casanare (Colombia), which is characterized by a savanna and gallery forest adjacent to water bodies. In this region, *Elaeis guineensis* plantations have mainly replaced the savanna that was previously used for cattle and agriculture activities. La Candelaria covers an area of 40 ha, where palms were sampled for triatomines in a gallery forest fragment (10 ha) and an *E. guineensis* plantation (24 ha). Los Potrillos is adjacent to the Cusiana River and comprises an area of 25 ha. Here, triatomines and small mammals were sampled in a gallery forest fragment (2-ha *Attalea butyracea* patch) and an *E. guineensis* plantation (11 ha). A pen, with numerous pigs (∼10), was located in the middle of the gallery forest fragment, and three dogs were found in the house. Black dots represent the locations of sampled palms, and circled numbers illustrate mammal sampling points as follows: 1) web sampling center for nonflying mammals and 2) mist-net locations for bat sampling. In both study sites, the brown-colored areas represent the domestic habitat in which plantation workers lived (∼5–10 people). This figure appears in color at www.ajtmh.org.

Fieldwork was conducted in the municipality of Tauramena (Casanare, Colombia) located in the Orinoco region, known as a high transmission area for Chagas disease.^15^ This region is characterized by savannas and gallery forests that form vegetation corridors along water bodies. The region has bimodal seasonality: the dry season occurs from November to March, and the rainy season between April and October.^16^ Tauramena has a mean annual temperature of 25°C and a mean annual precipitation of 2,400 mm.^16^ Sampling was performed in two study sites located 35 km apart, La Candelaria (LC) (4°40′13″N, 72°34′33″W) and Los Potrillos (LP) (4°59′1″N, 72°36′36″W). These sites were selected to consider two different landscapes: LC is located in the plains of the Orinoco region (∼50 km from the Andean Mountain piedmont) and LP is located closer to the Andean Mountain piedmont (∼25 km) (Figure 1). Human density in the Orinoco region is very low (less than 1 inhabitant per square kilometer).

The LC study site is composed of a gallery forest fragment (10 ha) with *A. butyracea* palms and an adjacent 14-year-old *E. guineensis* plantation (24 ha). *Attalea butyracea* density in LC is low (∼3 palms per ha), and the gallery forest in this site is composed of many tree species, from seedlings to adults. In addition, there is a low-flow stream (< 1 m^3^/s) across the gallery forest. Palms in both habitats were sampled in May, July, and December 2015, and March 2016.

Los Potrillos is located nearby the Cusiana River, an important river in the region, with an average flow of 200 m^3^/s. The study site comprises *A. butyracea* palms in a gallery forest fragment (2 ha) and a 10-year-old *E. guineensis* plantation (11 ha). The gallery forest in this site has only a few tree species seedlings, with *A. butyracea* being the predominant plant species (∼43 palms per ha) possibly because of landowners’ preferences. In addition, LP has early cacao crops adjacent to the gallery forest. Both habitats were sampled in August and December 2016, and March and July 2017.

Overall, each study site was sampled for 40 days: 10-day visits were performed four times per study site; thus, each site was visited twice per season. Each habitat (forest versus plantation) was sampled for five consecutive nights. *Elaeis guineensis* palms in both sites were harvested once every week and to the authors’ knowledge, workers lived in the dwellings next to the plantations (Figure 1).

### Triatomine sampling.

Triatomines were sampled between 17:00 hours and 07:00 hours, using live baited traps as described by Angulo et al.^17,18^ which had an approximate size of 30 × 20 × 25 cm and were baited with an adult chicken. Traps were set inside the palm crowns. All available *A. butyracea* were sampled (*N* = 101; 22 from LC and 79 from LP), whereas for *E. guineensis*, a subsample was selected based on the crown height (*N* = 189; 86 from LC and 103 from LP). Thus, 5% (∼86/1,800 palms) of the grove in LC and 13% (∼103/800 palms) in LP were sampled. The height was a major constrain for *E. guineensis* sampling; therefore, palm crowns were reached using a 10-m ladder. Some palm trees were 15 m tall, especially in LC, where the mean height was 12 m; the mean height of *E. guineensis* in LP was 6 m. *Attalea butyracea* palms in both study sites had a mean height of 12 m, which were reached using a ladder or a rope. *Elaeis guineensis* palms could not be reached using the rope because palm leaves and the distance between palms did not enable rope attachment.

Palms were geo-referenced and marked using aerosol spray paint. Collected triatomines were placed in 70% ethanol and transported to the laboratory at Universidad de los Andes for identification and further molecular procedures.

### Small mammal sampling.

**Figure 2.**
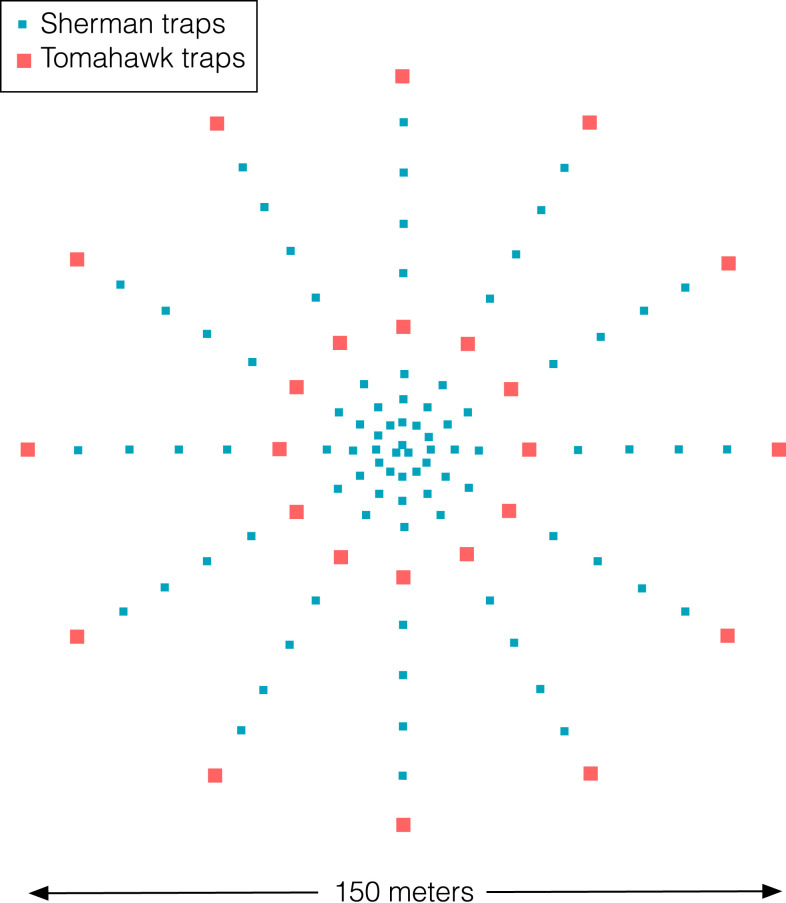
Arrangement of small mammal traps. Following a web sampling design, 111 traps were arranged for capturing non-volant mammals (87 Sherman, 24 Tomahawk). Twelve concentric transects were considered, with nine traps each and three Sherman traps in the middle of the web. Moving from the web center to the periphery, the first three traps were Sherman, the forth and ninth were Tomahawk, and the remaining were Sherman. The distance between the first three traps was 5 m and rest was 10 m. The web sampling design encompassed an area of 1.77 ha (radius 75 m^2^). This figure appears in color at www.ajtmh.org.

Mammals were sampled using 111 traps (87 Sherman, 24 Tomahawk) following a web sampling design^19^ to capture nonflying mammals. Sampling points were distributed in 12 concentric transects with nine traps each. In all transects, moving from the web center to the periphery, the first three traps were located every five meters, and the distance between the following six traps was 10 m. Tomahawk traps were located in the fourth and ninth positions, and the rest were Sherman traps. In addition, three Sherman traps were placed at the web center (Figure 2). All traps were baited daily at 18:00 hours using a mixture of hazelnut butter, oat, banana, and albacore and checked the next day at 07:00 hours. This procedure was conducted for five consecutive nights per habitat. Bats were captured using two mist nets set between 18:00 hours and 20:00 hours for three consecutive nights in each habitat for each of the four field trips per site.

Capture sites were geo-referenced, and animals were transported to the field laboratory for blood collection. Collected mammals were anesthetized using Zoletil^®^ (Virbac Laboratories, Carros, France [also known as Telazol^®^, Fort Dodge Animal Health, Fort Dodge, Iowa]) (0.05 mL/40 g) and bled by venipuncture using 0.5 mL sodium citrate. Blood samples were placed in guanidine hydrochloride buffer (2:1 volume of blood collected) and stored at room temperature. Captured mammals were sexed, measured, and photographed to further support species identification. Nonflying mammals were ear tagged for recapture analysis.

An additional field trip to LP was conducted in February 2018 to obtain blood samples from dogs (*Canis lupus familiaris*) and pigs (*Sus scrofa*). Pigs were located in the middle of the gallery forest at least 100 m away from the nearest *E. guineensis* plantation border. Dogs were captured from houses, at least 50 m from the gallery forest border and 60 m from the plantation. Domestic mammals were bled by venipuncture using 0.5 mL sodium citrate. For dogs, blood samples from the cephalic vein were obtained, and pigs were bled from the peripheral ear vein. Approximately 2.5 mL of blood were taken per domestic animal. Samples were placed in guanidine hydrochloride buffer (2:1) and stored at room temperature.

### *Trypanosoma cruzi* detection.

Only triatomine nymphs were dissected for further molecular analyses; adult triatomine dispersal hinders identification of their palm of origin. Therefore, the abdominal content of the nymph was dissected, macerated, and digested overnight using Proteinase K for DNA extraction preparation. Mammal blood samples were also prepared using digest buffer and Proteinase K. Then DNA was extracted using phenol–chloroform–isoamyl alcohol and reconstituted in 30 µL of TLE. *Trypanosoma cruzi* DNA was amplified using kDNA minicircle specific primers 121 (5′-AAATAATGT ACGG(T/G)GAGATGCATGA-3′) and 122 (5′GGGTTCGATTGGGGTTGGTGT-3′) to obtain an amplicon of 330 bp.^20^

### Triatomine and small mammal identification.

Triatomine species identification was carried out both by morphological and molecular analysis. Morphological identification was based on the Lent and Wygodzinsky^21^ taxonomic criteria. In addition, DNA samples of four triatomines (of 170) from LC and 16 triatomines (of 1,282) from LP were randomly selected for *cytb* gene amplification using primers cytb7432F (5′-GGACG(AT)GG(AT)ATTTATTATGGATC-3′) and cytb7433R (5′-GC(AT)CCAATTCA(AG)GTTA(AG)TAA-3′)^22^ to obtain a 682-bp DNA fragment. Polymerase chain reaction (PCR) products were sequenced using forward and reverse primers at the sequencing laboratory of Universidad de los Andes. The results were edited in Sequencher^®^ version 4-2.1.4 (Gene Codes Corporation Ann Arbor, MI) and analyzed using the online BLAST web interface provided by NCBI (http://blast.ncbi.nlm.nih.gov).

For sylvatic mammal species identification, barcoding analyses were performed in blood samples with sufficient volume using two pairs of primers. For LC samples, VerU-1 (f) (5′- AAGACGAGAAGACCCYATGGA-3′) and VerU-2 (r) (5′-CCTGATCCAACATMGAGGTCGTA-3′) primers^23^ were used to target the 16S ribosomal RNA region. The PCR products were sequenced with both forward and reverse primers at Sequencing Laboratory of Universidad de los Andes. The resulting sequences were edited using Sequencher^®^ version 4-2.1.4 (Gene Codes Corporation) and compared with in GenBank database (NCBI http://blast.ncbi.nlm.nih.gov). In LP, cytochrome c oxidase subunit I (COI) sequence primers (600–700 bp) for forensic barcoding were used.^24^ Unidirectional sequencing was performed by the CorpoGen-Universidad de los Andes Alliance. Identification was carried out according to the closest matching reference record in the Barcode of Life Data System (BOLD, www.boldsystems.org).

### Data analysis.

Data analyses were accomplished using R version 3.3.2^25^ and RStudio, an integrated development environment. Fisher’s exact test was used to compare palm tree infestation and *T. cruzi* infection in triatomines and small mammals between habitats. Welch two sample *t*-test was used to compare the triatomine density between *A. butyracea* and *E. guineensis* palms. The statistical tests were performed using “base” package, version 3.3.2.

A generalized linear model (GLM) was conducted to assess landscape configuration association with *T. cruzi* vector infection and abundance. This analysis was implemented at the individual palm tree level per habitat for each study site. The considered explanatory variables were palm distance (in kilometers) to the habitat centroid and to other habitat edges. To calculate distances, a raster of each study site segregated by habitats was created in ArcGIS 10.5, and centroids were estimated as the “most centered” pixel within a habitat raster. Thus, pixel *x* and *y* coordinates were calculated as the number of rows and columns, respectively, divided by two. Palm distances to habitat centroid and edges were calculated as the great circle distance between target pixel coordinates. For LC, the habitats considered were human settlements, forest, savanna, plantation, and shrub. For LP, the habitats considered were human settlements, forest, savanna, plantation, shrub, river, *A. butyracea* within forest fragments, and cacao crops. Vector abundance and infection of *T. cruzi* were the response variables; thus, two GLMs were performed per habitat. A quasi-binomial error distribution was implemented for *T. cruzi* vector infection because it is over-dispersed proportional data. For vector abundance, a quasi-Poisson error distribution was used because it is over-dispersed count data. Raster analysis was performed using “raster” package, version 2.6-7, and GLMs were conducted using “stats” package, version 3.4.3.

Spatial aggregation of triatomines was tested using Moran’s I index “ape” package for spatial autocorrelation in the *A. butyracea* and *E. guineensis* habitats (*P-*value *<* 0.05).^26^ Triatomine spatial distribution patterns were approached via interpolation analysis. For cross validation and predictions, a variogram to *R. prolixus* population density and palm coordinates was fitted and the geostatistical model defined. Interpolation analysis was performed using the autoKrige function, “automap” package.^27^

## RESULTS

### Triatomine sampling.

The overall sampling effort for triatomine searching in this study was 101 *A. butyracea* (LC: 22, LP: 79) and 189 *E. guineensis* (LC: 86, LP: 103) sampled palms. The proportion of triatomine-infested *A. butyracea* (72%) palms was significantly higher than *E. guineensis* palms in LP (50%) (Fisher’s exact test: *P-*value = 0.002, 95% CI: 1.44–Inf), but not different in LC (Fisher’s exact test: *P-*value *=* 0.12, 95% CI: 0.76–6.63) (Table 1). A total of 170 triatomines in LC and 1,282 in LP were captured using the baited traps. For LC, the average number of captured triatomines per infested palm was 2.73 (95% CI: 1.47–4.00) in *A. butyracea* and 5.61 (95% CI: 2.29–8.92) in *E. guineensis* plantation. This number was not significantly different between habitats (Welch two sample *t*-test—*t =* 1.69, *df =* 27.58, *P*-value = 0.10). For LP, the average number of captured triatomines per infested palm was 13.72 (95% CI: 9.61–17.83) in *A. butyracea* and 6.64 in *E. guineensis* (95% CI: 4.24–9.03). This number was significantly higher in *A.* butyracea than in *E. guineensis* (Welch two sample *t*-test—*t =* 2.974, df *=* 103.62, *P*-value = 0.004). The colonization index (palms with nymphs over positive palms) in *A. butyracea* was 0.95 in both study sites, and 1 and 0.95 in *E. guineensis* for LC and LP, respectively.

**Table 1:** Number of triatomines in positive palms (more than one triatomine) over total sampled palms in *Attalea butyracea* and *Elaeis guineensis* for La Candelaria and Los Potrillos sites, including statistical test results

	Positive palms*			Triatomines per positive palm†		
	*Attalea butyracea*	*Elaeis guineensis*	*P-*value‡	*Attalea butyracea*	*Elaeis guineensis*	*P-*value §
La Candelaria	10/22 (45%)	23/86 (27%)	0.12	2.73 (2.28)	5.61 (7.67)	0.10
Los Potrillos	57/79 (72%)	52/103 (50%)	0.002	13.72 (16.85)	6.64 (8.86)	0.004

* Infestation index is given in parentheses.

† Average triatomines per positive palm, and SD is given in parentheses.

‡ *P*-value from Fisher’s exact test.

§ *P*-value from the Welch two-sample *t* test.

**Figure 3.**
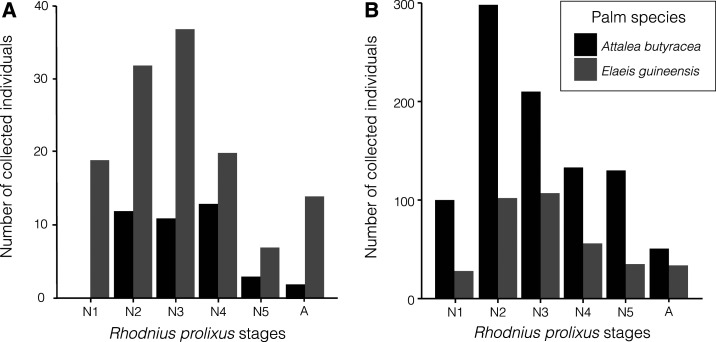
Number of collected *Rhodnius prolixus* classified by developmental stage. Histograms of collected *R. prolixus* in *Attalea butyracea* (black colored bars) and *Elaeis guineensis* (gray colored bars) palms are shown for La Candelaria (**A**) and Los Potrillos (**B**) study sites, categorized by nymphal stage: N1, N2, N3, N4, N5, and A for adult.

Based on morphology, all collected triatomines were identified as *R. prolixus*, and sequencing procedures confirmed these results for 14 triatomines (LC: 4, LP: 10) (lowest *E* values and sequence identity above 98%, GenBank codes: KP126725 to KP 126734). A higher number of nymphs than adults was collected in both palm species in the two study sites, with the intermediate nymph stages (II, III, IV) being the most abundant (Figure 3). No first stage nymphs were found in *A. butyracea* palms in LC (Figure 3A).

To determine *T. cruzi* infection in triatomines, 138 nymphs (Table 2) were analyzed in LC: 31 from *A. butyracea* and 107 from *E. guineensis*. Parasite prevalence was significantly lower in the *A. butyracea* palms (29%) than in the *E. guineensis* plantation (61%) (Fisher’s exact test: *P*-value *=* 0.002, 95% CI: 0–0.59). In LP, 165 nymphs (Table 2) from *A. butyracea* and 148 from *E. guineensis* were selected, for a total of 313 triatomines examined. Triatomine infection prevalence with *T. cruzi* was similar between habitats: 75% in the *E. guineensis* plantation and 76% in the *A. butyracea* forest (Table 2).

**Table 2:** Collected triatomines by stage and *Trypanosoma cruzi* infection in analyzed triatomine nymphs in *Attalea butyracea* and *Elaeis guineensis* palms in La Candelaria and Los Potrillos sites

		*Attalea butyracea*			*Elaeis guineensis*		
Study site	Stage	No.	*T. cruzi* (%)	Positive/analyzed	No.	*T. cruzi* (%)	Positive/analyzed
La Candelaria	N1	0	–	–	19	38	6/16
	N2	12	27	3/11	32	54	15/28
	N3	11	13	1/8	37	65	22/34
	N4	13	40	4/10	20	76	16/21
	N5	3	50	1/2	7	75	6/8
	Adult	2	–	–	14	–	–
	Total	41	29	9/31	129	61	65/107
Los Potrillos	N1	100	50	1/2	28	67	2/3
	N2	298	79	15/19	102	76	13/17
	N3	210	72	28/39	107	69	36/52
	N4	133	88	29/33	56	74	31/42
	N5	130	74	53/72	35	85	29/34
	Adult	50	–	–	33	–	–
	Total	921	76	126/165	361	75	111/158

N1–N5 = nymphs from the first to the fifth stage; no. = number of collected triatomines; positive/analyzed = number of *T. cruzi*–positive triatomines over analyzed; *T. cruzi* = percentage of infected individuals positive for *Trypanosoma cruzi*.

### Mammal sampling.

Nonflying mammals capture effort was 2,220 trap-nights per habitat (111 traps for five nights in four field trips), and capture effort for bats was 24 mist-net hours per habitat. In LC, 121 mammals were collected, 98 nonflying mammals (25 opossums and 73 rodents), and 23 bats (Table 3). Whereas rodents and opossums were captured in the gallery forest, only rodents were captured in *E. guineensis* plantations. Bats were captured in both habitats and belonged to two families, Phyllostomidae and Vespertilionidae.

**Table 3:** Comparative small mammal capture and *T. cruzi* infection in *Attalea butyracea* and *Elaeis guineensis* plantation in La Candelaria and Los Potrillos

		La Candelaria				Los Potrillos			
		*A. butyracea* patch		*E. guineensis* plantation		*A. butyracea* patch		*E. guineensis* plantation	
Mammal order	Scientific name	No.	*T. cruzi* (%)	No.	*T. cruzi* (%)	No.	*T. cruzi* (%)	No.	*T. cruzi* (%)
Didelphimorphia	*Didelphis marsupialis*	2	100	0	–	6	33.3	1	0
	*Marmosa* sp.	22	23	0	–	13	7.7	0	
	Total	24	29	0	–	19	15.8	1	0
Rodentia	*Holochilus sciureus*	–	–	–	–	1	0	0	–
	*Microryzomys minutus*	–	–	–	–	3	33.3	2	0
	*Mus musculus*	–	–	–	–	4	25	8	0
	*Oecomys trinitatis*	–	–	–		3	33.3	4	0
	*Oligoryzomys fulvescens*	–	–	–	–	5	0	14	0
	*Sigmodon alstoni*	–	–	–	–	3	66.6	2	0
	*Thomasomys laniger*	–	–	–	–	1	0	0	–
	*Zygodontomys brevicauda*	–	–	–	–	9	22.2	13	15
	Unidentified	13	7	53	23	8	0	24	0
	Total	13	7	53	23	37	18.9	67	3
Chiroptera	*Artibeus planirostris*	–	–	–	–	0	–	3	0
	*Artibeus* sp.	1	0	0	0		–		–
	*Carollia brevicauda*	–	–	–	–	0	–	4	0
	*Carollia perspicillata*	8	0	1	0	1	0	10	0
	*Carollia* sp.	–	–	–	–	3	33.3	2	0
	*Desmodus rotundus*	0	–	1	0	2	0	0	
	*Lophostoma brasiliense*	–	–	–	–	0	–	1	0
	*Molossus molossus*	–	–	–	–	0	–	3	0
	*Molossus rufus*	–	–	–	–	2	100	0	–
	*Myotis riparius*	2	0	0	–		–		–
	*Myotis* sp.	–	–	–	–	2	0	0	–
	*Noctilio leporinus*	–	–	–	–	0	–	1	0
	*Phyllostomus discolor*	–	–	–	–	0	–	1	0
	*Phyllostomus hastatus*	0	–	2	0				–
	Family Phyllostomidae	2	0	4	50	9	11.1	18	22.2
	*Platyrrhinus brachycephalus*	0	–	1	0		–		–
	*Saccopteryx bilineata*	–	–	–	–	1	0	2	50
	*Tonatia* (*Lophostoma*) *brasiliense*	1	0	0	–		–		–
	Total	14	0	9	22	20	20	45	11.1
Total small mammals		51	15.7	62	22.6	76	18.4	113	6.2

*A. butyracea* = *Attalea butyracea*; *E. guineensis* = *Elaeis guineensis*; *T. cruzi* = *Trypanosoma cruzi*.

In LP, 189 sylvatic mammals were captured: 124 nonflying mammals and 65 bats. Nonflying mammals included 20 opossums and 104 rodents from at least 10 genera or species (Table 3). Most opossums were captured in *A. butyracea* and the adjacent gallery forest (*n* = 19), except one *Didelphis marsupialis* that was found in the *E. guineensis* plantation. Overall, 35.5% (37/104) of all rodents were captured in the forest and 64.5% (67/104) in the *E. guineensis* plantation. A total of 65 bats from at least 13 genera or species were captured, from which 31% (20/65) were from *A. butyracea* and 69% (45/65) from the *E. guineensis* plantation.

The amplification and sequencing of the 16S ribosomal RNA region in mammals collected in LC were only successful for identifying some bat species (Table 3). On the other hand, COI primers allowed identification of mammals from bat, opossum, and rodent species in LP (Table 3), allowing comparison of mammalian diversity between habitats using a sample size–based rarefaction and extrapolation curves (Supplemental Material).

In LC, 113 mammals were analyzed for *T. cruzi* infection, 24 belonging to the order Didelphimorphia, 66 from Rodentia, and 23 from Chiroptera. In the gallery forest fragment, eight of 51 small mammals (seven opossums and one rodent) were positive for *T. cruzi*, with an overall prevalence of 15.69%. In the *E. guineensis* plantation, 23% of rodents (12 of 53) and two Phyllostomidae (cf. *Phyllostomus hastatus*) bats were infected with *T. cruzi*. The overall *T. cruzi* infection prevalence in *E. guineensis* plantation was 22.58% (Table 3). Small mammal infection with *T. cruzi* was not significantly different between habitats (Fisher’s exact test: *P*-value *=* 0.877). In LP, 11.11% of small mammals (21/189) were positive for *T. cruzi*. Small mammal infection prevalence with *T. cruzi* was significantly higher in the *A. butyracea* forest (18.4%) than in the *E. guineensis* plantation (6.2%) (Fisher’s exact test: *P*-value *=* 0.009, OR = 3.39).

Two dogs and three domestic pigs were sampled in LP, and one of each was positive for *T. cruzi.*

### Spatial analyses.

Generalized linear models showed that in LC, the distance from palms to the habitat center (measured as the centroid) or other habitat edges was not associated with vector abundance or infection with *T. cruzi* (coefficients *P* > 0.05). On the other hand, in LP, *T. cruzi* vector infection in *A. butyracea* was positively associated with proximity to cacao crops (GLM estimate −327.33, *t =* −2.085, *P*-value *=* 0.041) and vector abundance was negatively associated with human settlement proximity (GLM estimate −111.07, *t =* 2.141, *P*-value *=* 0.036). Vector abundance in the *E. guineensis* plantation was positively associated with proximity to the forest (GLM estimate −50.56, *t =* −2.265, *P*-value *=* 0.026) and to shrub (GLM estimate −42.10, *t =* −2.447, *P*-value *=* 0.016).

**Figure 4.**
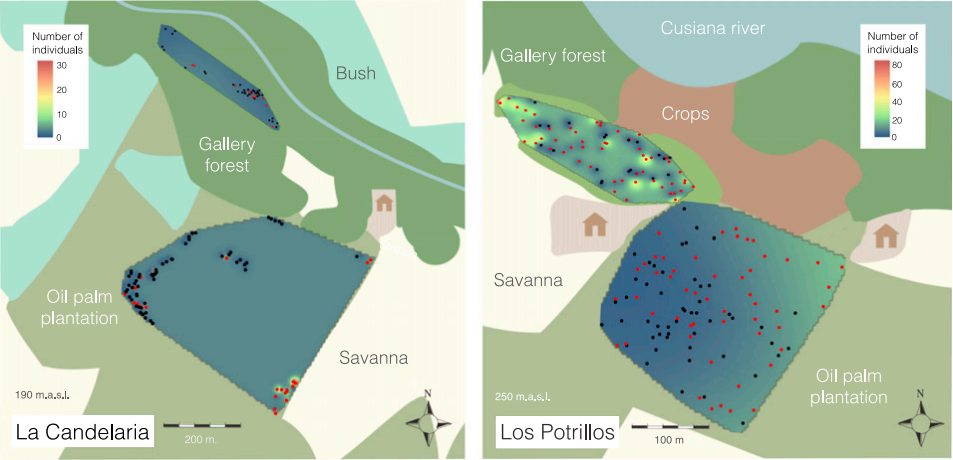
*Rhodnius prolixus* abundance interpolation. Areas showing high number of triatomines are colored from yellow to red and the lowest are blue colored (zero triatomines per palm). Dots represent the location of sampled palms. Black-colored dots are negative palms (no triatomines), and red-colored dots are positive palms (one or more triatomines). This figure appears in color at www.ajtmh.org.

The predicted triatomine distribution pattern in LC was clustered for the plantation (Moran’s I index *=* 0.27, *P*-value *=* 0.000), and the maximum estimated value per palm was 26.9 triatomines, located at the lower right corner next to the plantation and savanna (Figure 4). By contrast, for the gallery forest, triatomines were randomly distributed (Moran’s I index = −0.034, *P*-value *=* 0.760) and the maximum predicted value was 1.7 triatomines per palm.

In LP, triatomines were randomly distributed in *A. butyracea* palms (Moran’s I index = 0.03, *P*-value *=* 0.09) but not in the *E. guineensis* plantation (Moran’s I index = 0.04, *P*-value *=* 7 × 10^−5^). The predicted population relative density in *E. guineensis* plantation showed increases toward the corner connected to a gallery forest and an adjacent *E. guineensis* plantation, with a maximum estimated value of 13.5 triatomines per palm. The population density of triatomines across the *A. butyracea* forest displayed a randomly distributed pattern, and the highest triatomine density (88.9 triatomines per palm tree) was located at the westernmost point (Figure 4).

## DISCUSSION

In this study, the ability of *R. prolixus* to establish populations in *E. guineensis* plantations was confirmed by the presence of all nymphal stages in palm crowns. Multiple *Rhodnius* species, such as *Rhodnius ecuadoriensis*, *Rhodnius pictipes*, and *Rhodnius robustus*, have been reported in *E. guineensis* plantations in Ecuador.^28,29^ In addition, *Rhodnius pallescens* individuals have been found in the American oil palm (*Elaeis oleifera*) around the Colombia–Panama border.^30^ The results shown here corroborate the ability of *R. prolixus* to adapt to environmental changes and exploit new habitats.^31,32^

Triatomine sampling effort between habitats and study sites was heterogeneous because of logistical aspects, including accessibility to palm crowns, amount of palms, and seasonal variation. In both study sites, *A. butyracea* had higher *R. prolixus* infestation indices than *E. guineensis*. Previous studies in the area are scarce. One of the few projects that studied the role of *E. guineensis* plantations in *T. cruzi* transmission revealed an infestation index of 47% in *E. guineensis* palms by *R. prolixus*.^8^ The results shown here are comparable but varied between sites probably because of human intervention in LP gallery forest, leading to a higher *A. butyracea* density. In addition, in both study sites, *E. guineensis* plantations were harvested once per week and pesticide was frequently applied, potentially affecting the triatomine presence and abundance.

Studies on triatomine population density and distribution conducted by Urbano et al.^33^ in an *A. butyracea* forest and Suarez-Davalos et al.^34^ in sylvatic areas near dwellings predicted that the highest triatomine population was distributed toward domestic areas. By contrast, the spatial analyses presented here suggest that the triatomine relative population density in the *E. guineensis* plantation increased away from the human settlement to the gallery forest in LP (Figure 4). Triatomine abundance clustering in LC plantation had a similar pattern but toward the savanna and an adjacent plantation (Figure 4). The ongoing colonization process of *Rhodnius prolixus* and a possible edge effect^35^ could explain this disposition pattern because the high population densities in the *E. guineensis* plantation are bordering habitats where triatomines could come from. On the other hand, triatomine distribution in *A. butyracea* in LP was homogeneous (Figure 4) and negatively associated with the distance to the human settlement. These findings could be partially explained by a pig pen, located in the middle of the *A. butyracea* patch, and opossums presence, both providing a significant food source.^36,37^ Although it was not a part of this study, high triatomine relative abundance could be correlated to the presence of abundant blood sources as was demonstrated by Gurgel-Gonçalves et al.^38^ who showed that relative abundance of two triatomine species (*Rhodnius neglectus* and *Psammolestes tertius*) was greater in areas with a higher number of bird nests in palms. Birds, despite not being of epidemiological importance as *T. cruzi* reservoirs, do act as a blood source for triatomines, and palms can provide them nesting opportunities in a highly anthropogenically altered landscape.

High infection prevalence with *T. cruzi* found in LP could be strongly related to neighboring triatomine habitats, such as *A. butyracea* palms located in gallery forests, pastures, and human settlements.^39^ *Trypanosoma cruzi* natural infection prevalence in *R. prolixus* collected in *E. guineensis* in both study sites was similar to previous reports in *A. butyracea* in the department of Casanare, reaching 85%.^7,33^ This suggests that *T. cruzi* transmission in the region is stable, and in this context, oil palm plantations are rapidly becoming extensions of *A. butyracea* areas.

The potential for the establishment of a transmission cycle in *E. guineensis* plantations was evaluated by sampling small mammals. Comparing the *E. guineensis* plantation with the *A. butyracea* patch, opossums were less captured than rodents in the plantation in both study sites. Because most of captured opossums were *Marmosa* sp., which are known as forest specialists,^40^ their absence in the plantations is reasonable. On the other hand, captured rodent species are generalists and terrestrial (*Mus musculus*, *Zygodontomys brevicauda*, and *Oligoryzomys fulvescens*),^41^ thus abundant in both habitats. Generalist host species, such as opossums and rodents, have been previously associated with high triatomine infection by *T. cruzi*.^42,43^ Finally, *R. prolixus* infection with *T. cruzi* in LP increased with proximity to the cacao crop, which could be explained by the fact that rodents are attracted to cacao beans for consumption.

The composite prevalence of *T. cruzi* infection across small mammal hosts is comparable with values reported in the region.^7^ Infected mammals in both habitats were predominantly rodents, suggesting them as important hosts in both *T. cruzi* transmission cycles. Notably, in LP, the proportion of infected *R. prolixus* was similar in both habitats, but triatomine abundance was higher in *A. butyracea* palms, increasing the overall vector–host contact rate and theoretically leading to the higher observed infection prevalence in small mammals with *T. cruzi*. This finding suggests that oil palm plantations could be sustaining an overall higher *T. cruzi* infection prevalence by harboring mostly generalist rodents. Mathematical modeling could be useful for studying this hypothesis.

Our findings also suggest that *T. cruzi* transmission in LP may be partly maintained by *T. cruzi* infection in pigs and dogs. Dogs have been reported as important host sentinels in many transmission scenarios.^44^ Previous studies in Colombia have shown a *T. cruzi* infection prevalence in dogs between 50% and 70.1% in the Caribbean region,^45,46^ 31% in the department of Boyacá,^47^ and 8.3% in the department of Casanare.^48^ The role of domestic pigs in the *T. cruzi* transmission cycle in Colombia remains poorly studied, as only one study has reported *T. cruzi* infection in one sampled individual.^45^ Nonetheless, in other endemic regions, such as the Brazilian Amazon Basin, pig pens adjoining human dwellings were infested with *Panstrongylus geniculatus*, and sampled domestic pigs were infected.^36^ The role of domestic pigs in Chagas disease infection risk requires further research.

Transmission scenarios vary depending on ecological, meteorological, epidemiological, and socioeconomic conditions. For instance, Rendon et al.^7^ reported a robust sylvatic cycle in a rural area located in the municipality of Mani (Casanare), with the absence of triatomines in houses and *T. cruzi* infection in sylvatic mammals. However, Angulo-Silva et al.^48^ suggested that the *T. cruzi* sylvatic cycle was extended to human dwellings in the same municipality. Other studies in the northeast of Colombia have indicated that sylvatic and domestic cycles do not overlap.^49^ Our study suggests a *T. cruzi* transmission scenario with a high *T. cruzi* natural infection prevalence in triatomines that could be maintained by both sylvatic and domestic mammals, but the precise contribution of each remains to be ascertained. A similar scenario of transmission was reported in Sierra Nevada de Santa Marta indigenous communities where authors reported overlapping domestic and sylvatic cycles of *T. cruzi*.^45^

This study did not account for temperature or relative humidity in *A. butyracea* nor *E. guineensis* palms; however, microclimate within palm crowns could be an important factor driving *R. prolixus* abundance as has been suggested by previous studies.^33^ In addition, seasonality, an important variable driving populations and consequently transmission, was out of the study scope. Sylvatic non-volant mammals were sampled using Sherman and Tomahawk traps, allowing the capture of rodents and small-sized opossums. However, small mammal sampling effort in this study did not allow determination of their abundance, and this can always lead to biased results. In addition, to fully comprehend the sylvatic transmission cycle, the capture of medium- to large-sized mammals and xenodiagnoses experiments to determine the relative reservoir competence are required. Mammal traps are more efficient in resource-limited habitats, and results such as opossum absence in the *E. guineensis* plantations could also be related to insufficient resources in this habitat. Finally, a potential bias could be present because PCR products from *T. cruzi* infection analysis were not sequenced, and most of the analyzed triatomines corresponded to fourth and fifth nymphal stages.

In summary, *E. guineensis* plantations in the department of Casanare are suitable habitats for *R. prolixus* and *T. cruzi* transmission. The parasite transmission scenario in *E. guineensis* plantations is comparable with adjacent anthropogenically altered habitats where *A. butyracea* palms are present, and the *T. cruzi* transmission cycle is maintained by domestic and sylvatic mammal hosts. Disease transmission dynamics respond to environmental changes in complex ways, and the effects of land-use alteration require very precise knowledge.^12^ *Elaeis guineensis* plantation establishment in the Orinoco region represents an important risk factor for Chagas disease because it increases the distribution of *R. prolixus*.^10,11^ Moreover, a significant area of current *E. guineensis* plantations in the Orinoco region replaced pastures and savannas, resulting in an enormous land use change in the region. Finally, *E. guineensis* plantations in the department of Casanare are often established near human dwellings to facilitate plantation worker accessibility, bringing vectors into close contact with people and increasing the risk of infection with *T. cruzi*. Further studies on these new transmission scenarios of rapid land-use changes are encouraged.

## Supplemental material

Supplemental materials

## References

[1] World Health Organization, 2010 WHA63.20. WHO Eighth Plentary Meeting, 21 May 2010, Committee A, Fifth Report. Geneva, Switzerland: WHO.

[2] Rassi A Jr. Rassi A Marin-Neto JA, 2010 Chagas disease. Lancet 375: 1388–1402. 20399979 10.1016/S0140-6736(10)60061-X

[3] Moncayo A Silveira AC, 2009 Current epidemiological trends for Chagas disease in Latin America and future challenges in epidemiology, surveillance and health policy. Mem Inst Oswaldo Cruz 104 (Suppl 1): 17–30. 19753454 10.1590/s0074-02762009000900005

[4] Guhl F 1999 Estado actual del control de la enfermedad de chagas en Colombia. Medicina (B Aires) 59 (Suppl 2): 103–116. 10668251

[5] Abad-Franch F 2015 On palms, bugs, and Chagas disease in the Americas. Acta Trop 151: 126–141. 26196330 10.1016/j.actatropica.2015.07.005

[6] Sanchez-Martin MJ Feliciangeli MD Campbell-Lendrum D Davies CR, 2006 Could the Chagas disease elimination programme in Venezuela be compromised by reinvasion of houses by sylvatic *Rhodnius prolixus* bug populations? Trop Med Int Health 11: 1585–1593. 17002733 10.1111/j.1365-3156.2006.01717.x

[7] Rendon LM Guhl F Cordovez JM Erazo D, 2015 New scenarios of *Trypanosoma cruzi* transmission in the Orinoco region of Colombia. Mem Inst Oswaldo Cruz 110: 283–288. 25830543 10.1590/0074-02760140403 PMC4489465

[8] Guhl F Pinto N Marín D Herrera C Aguilera G Naranjo J Vallejo G, 2005 Primer reporte de Rhodnius prolixus Stal, en Elaeis guineensis variedad Papúa, en plantaciones agroindustriales de Villanueva, Casanare. Biomédica 25: 158–159.

[9] Henson IE Romero RR Romero HM, 2011 The growth of the oil palm industry in Colombia. J Oil Palm Res 23: 1121–1128.

[10] Castiblanco C Etter A Aide TM, 2013 Oil palm plantations in Colombia: a model of future expansion. Environ Sci Pol 27: 172–183.

[11] Cordovez JM Guhl F, 2015 The impact of landscape transformation on the reinfestation rates of *Rhodnius prolixus* in the Orinoco region, Colombia. Acta Trop 151: 73–79. 26254003 10.1016/j.actatropica.2015.07.030

[12] Gottdenker NL Streicker DG Faust CL Carroll CR, 2014 Anthropogenic land use change and infectious diseases: a review of the evidence. Ecohealth 11: 619–632. 24854248 10.1007/s10393-014-0941-z

[13] Kwa BH, 2008 Environmental change, development and vectorborne disease: malaysia’s experience with filariasis, scrub typhus and dengue. Environ Dev Sustain 10: 209–217.

[14] Pluess B Mueller I Levi D King G Smith TA Lengeler C, 2009 Malaria–a major health problem within an oil palm plantation around Popondetta, Papua New Guinea. Malar J 8: 56. 19356235 10.1186/1475-2875-8-56 PMC2682491

[15] Guhl F Aguilera G Pinto N Vergara D, 2007 Actualización de la distribución geográfica y ecoepidemiología de la fauna de triatominos ( Reduviidae: Triatominae ) en Colombia. Biomédica 27: 143–162. 18154255

[16] Minorta-Cely V Rangel-Churio JO, 2014 El clima de la Orinoquia colombiana. Olombia Diversidad Biótica XIV. La Región De La Orinoquia De Colombia. Bogotá, Colombia: Instituto de Ciencias Naturales, Universidad Nacional de Colombia, 153–206.

[17] Angulo VM Esteban L, 2011 Nueva trampa para la captura de triatominos en hábitats silvestres y peridomésticos. Biomédica 31: 264–268. 22159544 10.1590/S0120-41572011000200015

[18] Angulo VM Esteban L Luna KP, 2012 Attalea butyracea próximas a las viviendas como posible fuente de infestación domiciliaria por *Rhodnius prolixus* (Hemiptera: Reduviidae) en los Llanos Orientales de Colombia. Biomédica 32: 277–285. 23242302 10.1590/S0120-41572012000300016

[19] Parmenter RR 2003 Small-mammal density estimation: a field comparison of grid-based vs. web-based density estimators. Ecol Monogr 73: 1–26.

[20] Britto C Cardoso MA Vanni CM Hasslocher-Moreno A Xavier SS Oelemann W Santoro A Pirmez C Morel CM Wincker P, 1995 Polymerase chain reaction detection of *Trypanosoma cruzi* in human blood samples as a tool for diagnosis and treatment evaluation. Parasitology 110 (Pt 3): 241–247. 7724232 10.1017/s0031182000080823

[21] Lent H Wygodzinsky P, 1979 Revision of the Triatominae (Hemiptera, Reduviidae), and their significance as vectors of Chagas disease. Bull Am Museum Nat Hist 163: 123–520.

[22] Fitzpatrick S Feliciangeli MD Sanchez-Martin MJ Monteiro Fa Miles Ma, 2008 Molecular genetics reveal that silvatic *Rhodnius prolixus* do colonise rural houses. PLoS Negl Trop Dis 2: e210. 18382605 10.1371/journal.pntd.0000210 PMC2270345

[23] Sawabe K 2010 Host-feeding habits of *Culex pipiens* and *Aedes albopictus* (Diptera: Culicidae) collected at the urban and suburban residential areas of Japan. J Med Entomol 47: 442–450. 20496592 10.1603/ME09256

[24] Ivanova NV Clare EL Borisenko AV, 2012;. DNA barcoding in mammals. Methods Mol Biol 858: 153–182. 22684956 10.1007/978-1-61779-591-6_8

[25] R Development Core Team, 2016 R: A Language and Environment for Statistical Computing. Vienna Austria: R Foundation for Statistical Computing.

[26] Paradis E Claude J Strimmer K, 2004 APE: analyses of phylogenetics and evolution in R language. Bioinformatics 20: 289–290. 14734327 10.1093/bioinformatics/btg412

[27] Hiemstra PH Pebesma EJ Twenhöfel CJW Heuvelink GBM, 2007 Automatic real-time interpolation of radiation hazards: a prototype and system architecture considerations. Int J Spat Data Infrastruct Res 3: 58–72.

[28] Cuba Cuba CA Abad-Franch F Rodríguez JR Vásquez FV Velasquez LP Miles MA, 2002 The triatomines of northern Peru, with emphasis on the ecology and infection by trypanosomes of *Rhodnius ecuadoriensis* (Triatominae). Mem Inst Oswaldo Cruz 97: 175–183. 12016438 10.1590/s0074-02762002000200005

[29] Abad-Franch F Palomeque FS Aguilar VHM Miles MA, 2005 Field ecology of sylvatic *Rhodnius populations* (Heteroptera, Triatominae): risk factors for palm tree infestation in western Ecuador. Trop Med Int Health 10: 1258–1266. 16359406 10.1111/j.1365-3156.2005.01511.x

[30] Jaramillo N Schofield CJ Gorla D Caro-Riaño H Moreno J Mejia E Dujardin JP, 2000 The Role of *Rhodnius pallescens* as a vector of Chagas disease in Colombia and Panama. Res Rev Parasitol 60: 75–82.

[31] Schofield CJ Galvão C, 2009 Classification, evolution, and species groups within the Triatominae. Acta Trop 110: 88–100. 19385053 10.1016/j.actatropica.2009.01.010

[32] Dias FBS Quartier M Diotaiuti L Mejía G Harry M Lima ACL Davidson R Mertens F Lucotte M Romaña CA, 2014 Ecology of *Rhodnius robustus* larrousse, 1927 (Hemiptera, Reduviidae, Triatominae) in Attalea palm trees of the Tapajós river region (Pará state, Brazilian Amazon). Parasit Vectors 7: 154. 24690302 10.1186/1756-3305-7-154 PMC3974420

[33] Urbano P Poveda C Molina J, 2015 Effect of the physiognomy of *Attalea butyracea* (Arecoideae) on population density and age distribution of *Rhodnius prolixus* (Triatominae). Parasit Vectors 8: 1–12. 25889617 10.1186/s13071-015-0813-6 PMC4389994

[34] Suarez-Davalos V Dangles O Villacis AG Grijalva MJ, 2010 Microdistribution of sylvatic triatomine populations in central-coastal Ecuador. J Med Entomol 47: 80–88. 20180312 10.1603/033.047.0111

[35] Fahrig L, 2003 Effects of habitat fragmention on biodiversity. Annu Rev Ecol Syst 34: 487–515.

[36] Valente VC Valente SAS Noireau F Carrasco HJ Miles MA, 1998 Chagas disease in the Amazon Basin: association of *Panstrongylus geniculatus* (Hemiptera: Reduviidae) with domestic pigs. J Med Entomol 35: 99–103. 9538568 10.1093/jmedent/35.2.99

[37] Noireau F Diosque P Jansen AM, 2009 *Trypanosoma cruzi*: adaptation to its vectors and its hosts. Vet Res 40: 1–23. 19250627 10.1051/vetres/2009009 PMC2695024

[38] Gurgel-Gonçalves R Duarte MA Ramalho ED Torre Palma AR Romaña CA Cuba-cuba CA, 2004 Spatial distribution of Triatominae populations ( Hemiptera: Reduviidae ) in *Mauritia flexuosa* palm trees in Federal district of Brazil. Rev Soc Bras Med Trop 37: 241–247. 15330065 10.1590/s0037-86822004000300010

[39] Gottdenker NL Calzada JE Saldaña A Carroll CR, 2011 Association of anthropogenic land use change and increased abundance of the Chagas disease vector *Rhodnius pallescens* in a rural landscape of Panama. Am J Trop Med Hyg 84: 70–77. 21212205 10.4269/ajtmh.2011.10-0041 PMC3005514

[40] Emmons L, 1990 Neotropical Rainforest Mammals. A Field Guide. Chicago, London: The University of Chicago Press.

[41] Suzán G Armién A Mills JN Marcé E Ceballos G Ávila M Salazar-Bravo J Ruedas L Armién B Yates TL, 2008 Epidemiological considerations of rodent community composition in fragmented landscapes in Panama. J Mammal 89: 684–690.

[42] Gottdenker NL Chaves LF Calzada JE Saldaña A Carroll CR, 2012 Host Life history strategy, species diversity, and habitat influence *Trypanosoma cruzi* vector infection in changing landscapes. PLoS Negl Trop Dis 6: 5–7. 10.1371/journal.pntd.0001884 PMC3499412 23166846

[43] Erazo D Cordovez J Cabrera C Calzada JE Saldana A Gottdenker NL, 2017 Modelling the influence of host community composition in a sylvatic *Trypanosoma cruzi* system. Parasitology 144: 1881–1889. 28701240 10.1017/S0031182017001287

[44] Cohen JE Gürtler RE, 2001 Modeling household transmission of American trypanosomiasis. Science 293: 694–698. 11474111 10.1126/science.1060638

[45] Mejía-Jaramillo AM Agudelo-Uribe LA Dib JC Ortiz S Solari A Triana-Chávez O, 2014 Genotyping of *Trypanosoma cruzi* in a hyper-endemic area of Colombia reveals an overlap among domestic and sylvatic cycles of Chagas disease. Parasit Vectors 7: 108. 24656115 10.1186/1756-3305-7-108 PMC3994407

[46] Cantillo-Barraza O Garcés E Gómez-Palacio A Cortés LA Pereira A Marcet PL Jansen AM Triana-Chávez O, 2015 Eco-epidemiological study of an endemic Chagas disease region in northern Colombia reveals the importance of *Triatoma maculata* (Hemiptera: Reduviidae), dogs and *Didelphis marsupialis* in *Trypanosoma cruzi* maintenance. Parasit Vectors 8: 482. 26394766 10.1186/s13071-015-1100-2 PMC4580378

[47] Ramírez JD Turriago B Tapia-Calle G Guhl F, 2013 Understanding the role of dogs (*Canis lupus* familiaris) in the transmission dynamics of *Trypanosoma cruzi* genotypes in Colombia. Vet Parasitol 196: 216–219. 23351975 10.1016/j.vetpar.2012.12.054

[48] Angulo-Silva VM Castellanos-Domínguez YZ Flórez-Martínez M Esteban-Adarme L Pérez-Mancipe W Farfán-García AE Luna-Marín KP, 2016 Human trypanosomiasis in the eastern plains of Colombia: new transmission scenario. Am J Trop Med Hyg 94: 348–351. 26728765 10.4269/ajtmh.15-0406 PMC4751963

[49] Pena-García VH Gómez-Palacio AM Triana-Chávez O Mejía-Jaramillo AM, 2014 Eco-epidemiology of chagas disease in an endemic area of Colombia: risk factor estimation, *Trypanosoma cruzi* characterization and identification of blood-meal sources in bugs. Am J Trop Med Hyg 91: 1116–1124. 25331808 10.4269/ajtmh.14-0112 PMC4257632

