# Supplementary material for: Rhodnius prolixus Colonization and Trypanosoma cruzi Transmission in Oil Palm (Elaeis guineensis) Plantations in the Orinoco Basin, Colombia
Source: Am J Trop Med Hyg. 2020 May 26;103(1):428–36. doi: 10.4269/ajtmh.19-0331 (PMC7356441; doi:10.4269/ajtmh.19-0331)
Supplement: Supplementary file 1 [file tpmd190331.SD1.docx]

**RAREFACTION ANALYSIS**

To compare species diversity of mammals between habitat types in Los Potrillos, a sample-size-based rarefaction and extrapolation curves were developed based on three measures of Hill numbers of order q: species richness (q = 0), Shannon diversity (q = 1, the exponential of Shannon entropy) and Simpson diversity (q = 2, the inverse of Simpson concentration) (Chao 2014, Hsieh et al. 2016). These analyses and a sample coverage estimation was also performed using iNEXT package (Hsieh et al. 2016) in R.

Confidence intervals (95%) in the rarefaction and extrapolation curves indicated significant differences in the species diversity of the assemblages of non-flying mammals between habitat types at Los Potrillos. On the contrary, the diversity of bats did not differ among habitat types, as revealed by the overlap of Hill numbers confidence interval. The sample coverage was higher than 90% for both habitat types.

The sampling effort allowed us to characterize the non-flying small mammal diversity in both habitat types. The lower species richness and diversity of non-flying small mammals in oil palm plantations could eventually be related with the higher prevalence of *T. cruzi* in these monocultures, as proposed by Xavier et al. (2012). A higher sampling effort is required for a comprehensive bat inventory at the study site; however, this data suggests that some phyllostomid could be involved in the dispersion of *T. cruzi* in the Orinoco region, Colombia. Considering bat dispersal, it is difficult to state with certainty whether an individual captured in one habitat type effectively roosts or forages there. Thus, testing this supposition requires the development of long term studies including capture/recapture data.


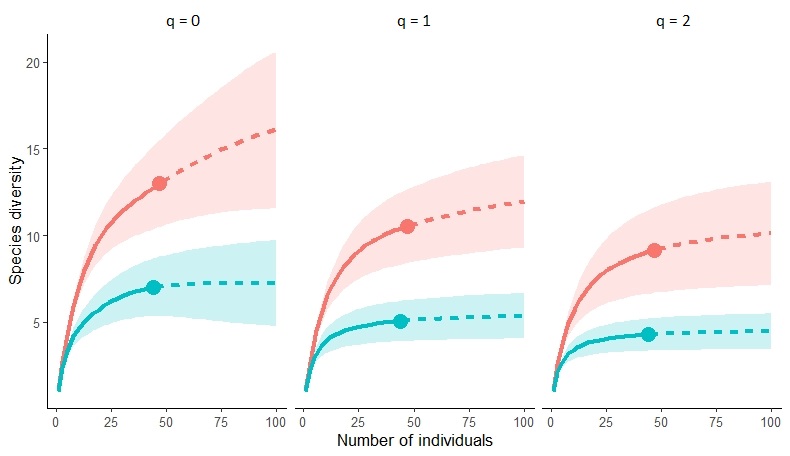


**Figure S1**. Sample-size-based rarefaction (solid lines) and extrapolation (dashed lines) curves for non-flying mammalian species diversity in secondary forests (red lines) and oil palm plantations (blue lines) at Los Potrillos. The species diversity in each panel correspond to species richness (q = 0), Shannon diversity (q = 1) and (c) Simpson diversity (q = 2). The 95% confidence intervals for species diversity in each habitat type are represented by the shaded areas. Reference samples are denoted by solid dots.


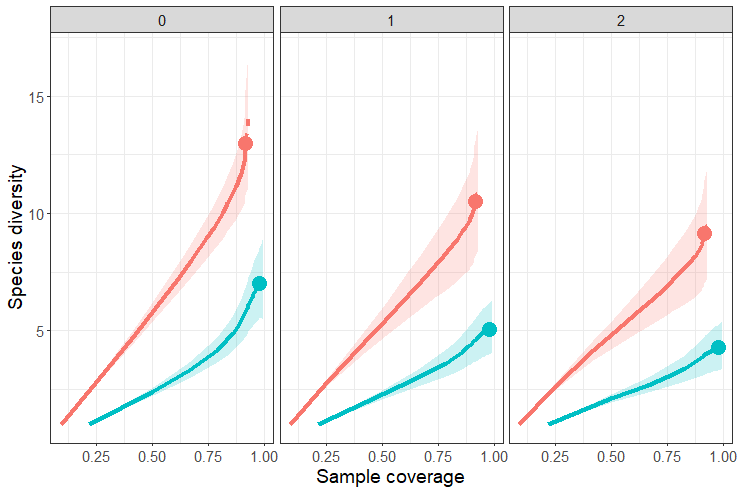


**Figure S2**. Sample coverage curves for non-flying mammalian species diversity in secondary forests (red lines) and oil palm plantations (blue lines) at Los Potrillos.

**References**

Chao A, Gotelli NJ, Hsieh TC, Sander EL, Ma KH, Colwell RK, Ellison AM. Rarefaction and extrapolation with Hill numbers: a framework for sampling and estimation in species diversity studies. Ecol. Monograph. 2014; 84(1): 45­-67. doi:10.1890/13-0133.1

Hsieh TC, Ma KH, Chao A. iNEXT: An R package for interpolation and extrapolation of species diversity (Hill numbers). Methods Ecol. Evol. 2016; 7: 1451-1456. doi:10.1111/2041-210X.12613

Xavier SCdC, Roque ALR, Lima VdS, Monteiro KJL, Otaviano JCR, Ferreira da Silva LFC, Jansen, AM. Lower Richness of Small Wild Mammal Species and Chagas Disease Risk. PLoS Negl. Trop. Dis. 2012; 6(5): e1647. https://doi.org/10.1371/journal.pntd.000164
